# Characterization of the complete mitochondrial genome of a nematode species, *Caenorhabditis tribulationis* (Nematoda, Rhabditidae)

**DOI:** 10.1080/23802359.2022.2079103

**Published:** 2022-06-02

**Authors:** Yuheng Liu, Zhang Jingjing, Chao Li

**Affiliations:** a Department of Microbiology and Biochemical Pharmacy, College of Pharmacy, National Engineering Research Center of Immunological Products, Third Military Medical University, Chongqing, P. R. China; b School of Pharmacy, Lanzhou University, Lanzhou, P. R. China

**Keywords:** *Caenorhabditis tribulationis*, mitogenome sequence, assembly, phylogenetic analysis

## Abstract

In the present study, we reported the complete mitogenome sequence of *Caenorhabditis tribulationis* Stevens & Félix 2019. The whole mitogenome of *C. tribulationis* is 14006 bp in length with an extreme bias of high AT content (75.26%) (GenBank accession no. OL362111). The mitochondrial genome contains 12 protein-coding genes (PCGs), 22 transfer RNA (tRNAs) genes, 2 ribosomal RNA (12S rRNA and 16S rRNA) genes, and a control region. All genes were unidirectionally transcribed on the same strand, typical for other nematode mitogenomes. 9 PCGs were initiated by typical ATN codons, except for NAD2, CYTB and NAD4, which were start with TTG codons. All the PCGs were predicted to use the typical TAN as the stop codons. The phylogenetic analysis showed that the relationship of *C. tribulationis* is very close to other species in the family Rhabditidae and separated form species of the families Ascarididae, Toxocaridae, Anisakidae and Ascaridiidae with high bootstrap value support.

Caenorhabditis species exist in soil, fresh water, sea water and other environments, and flourish in places with rich rotten plants or other microorganisms (Ferrari et al. 2017). Morphology is the most common method for species identification, however, closely related species are often morphologically very similar, which hinders the discovery of new Caenorhabditis species (Sudhaus and Kiontke 2007). Mitochondrial DNA (mtDNA) has been proved to be an effective method for species identification (Hu et al. 2003). In this study, the complete mitochondrial genome of *C. tribulationis* (Stevens et al. 2019)was recovered through Illumina sequencing data, and this complete mitochondrial genome can be subsequently used for testing the phylogeny relationships among Caenorhabditis species.

The adult female specimen of *C. tribulationis* was obtained from rotting fruit collected at Baiyin (36°33′N, 104°11′E), Gansu of China. The specimen was conserved in School of pharmacy, Lanzhou University under the accession number of No. BY201801 (contect person: Yuheng Liu, yhliu2017@lzu.edu.cn). Total genomic DNA was extracted using QIAGEN DNeasy Extraction Kit following the manufactures instructions. Whole-genome sequencing was performed on the Illumina HiSeq 2500 Sequencing Platform (Illumina, San Diego, CA, USA), and the complete mitochondrial genome were assembled using SPAdes 3.9.0 (Bankevich et al. 2012). The complete sequence was primarily annotated by MITOS WebServer (Bernt et al. 2013) and all the predicted tRNAs were confirmed using the tRNAscan-SE search server (Lowe and Chan 2016). Protein-coding genes (PCGs) and rRNA genes were annotated manually based on BLASTn results against published sequences of *C. brenneri* (GenBank: KY552900.1). The concatenated amino acid sequences of the 12 PCGs were used to reconstruct the phylogenetic relationships among the species within Ascarididae, Toxocaridae, Anisakidae, Rhabditidae and Ascaridiidae using the Maximum Likelihood (ML) algorithm in MEGA6.0 software with the Jones-Taylor-Thornton (JTT) mode, considering 2000 replications with bootstrap analyses (Tamura et al. 2013).

The complete mitochondrial genome of *C. tribulationis* is 14006 bp in length and has a base composition of A (29.32%), T(45.94%), C(15.36%), G(9.38%), demonstrating an extreme bias of high AT content (75.26%) (GenBank accession no. OL362111). The mitochondrial genome contains a typically conserved structure among nematode mitogenomes, encoding 12 protein-coding genes (PCGs), 22 transfer RNA (tRNA) genes, 2 ribosomal RNA (12S rRNA and 16 s rRNA) genes and a control region (D-loop region). The mitochondrial gene order was identical to that observed in most nematode genomes, and all genes were unidirectionally transcribed on the same strand. 9 PCGs were initiated by typical ATN codons (ATT for NAD6, NAD4L, NAD1, ATP6, COX1, COX2, NAD3 and NAD5; ATA for COX3), except for NAD2, CYTB and NAD4, which were start with TTG codons. All the PCGs were predicted to use the typical TAN as the stop codons. Most of the TΨC arm of tRNAs were replaced by the TV-replacement loop, only 3 tRNAs contained standard secondary structure (Wolstenholme et al. 1994). The phylogenetic analysis showed that the relationship of *C. tribulationis* is very close to other species in the family Rhabditidae and separated form species of the families Ascarididae, Toxocaridae, Anisakidae and Ascaridiidae with high bootstrap value support (Figure 1).

**Figure 1.**
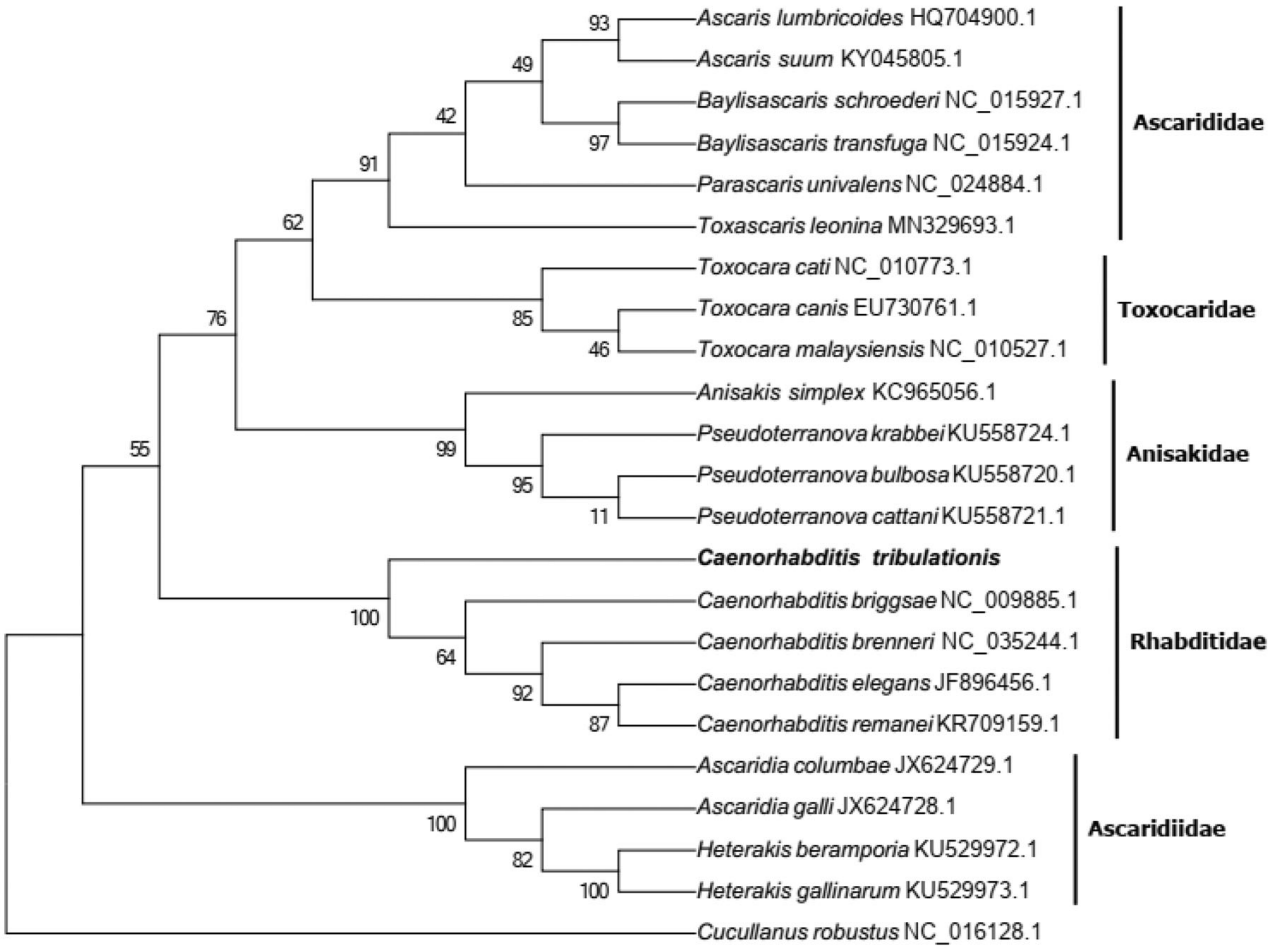
Phylogenetic tree constructed for 22 nematode species, including *C. tribulationis*, using the concatenated amino acid sequences of 12 PCGs. GenBank accession numbers of each species were listed in the tree. *Cucullanus robustu*s was used as the outgroup. The tree was constructed based on a complete protein sequence alignment by the ML method with 2000 bootstrap replications.

## Data Availability

The genome sequence data that support the findings of this study are openly available in GenBank of NCBI at (https://www.ncbi.nlm.nih.gov/) under the accession no. OL362111. The associated BioProject, SRA, and Bio-Sample numbers are PRJEB36817, ERR5967935, and SAMEA8556781 respectively.
